# The development and the use of gender-affirming online resources and games for gender-independent, intersex, non-binary, and transgender (GIaNT) children and youth: A scoping review protocol

**DOI:** 10.1371/journal.pone.0294869

**Published:** 2023-11-29

**Authors:** Roya Haghiri-Vijeh, Kat Newman-Seymour, Daniel Huizenga, Aidan Hung

**Affiliations:** 1 School of Nursing, Faculty of Health, York University, Toronto, ON, Canada; 2 Gender, Sexuality, Women’s Studies, Western University, London, ON, Canada; 3 Social Innovation Research Centre, Centennial College, Scarborough, ON, Canada; 4 School of Nursing, Collaborative Toronto Metropolitan University, Centennial and George Brown College Nursing Degree Program, Canada; University of Technology Sydney, AUSTRALIA

## Abstract

**Objective:**

The objective of this scoping review protocol is to review what has been reported on the development and the use of gender-affirming online resources and games for gender-independent, intersex, non-binary, and transgender (GIaNT) youth (aged 9–26).

**Introduction:**

GIaNT youth and their specialized health care needs are mostly exempt from curriculums. There is limited information on the specific online sources available for GIaNT children and youth.

**Inclusion criteria:**

The inclusion criteria are sources that include GIaNT children and youth and focus on online spaces and games for the identified population.

**Methods:**

The Joanna Briggs Institute (JBI) method for scoping reviews has guided the development of this protocol. Databases to be searched include CINAHL, Cochrane, Epistemonikos, ERIC, Gender Studies Database, GenderWatch, LGBTQ+ Source, ProQuest, PyscInfo, and Scopus. Unpublished studies and gray literature searches will be undertaken in ProQuest thesis and dissertation and a limited number of relevant websites. No limit on date or region will be applied. Records will be screened and extracted by two independent reviewers. Results will be presented as tables with accompanying narrative summary.

**Conclusion:**

This scoping review protocol will guide the review and mapping of literature on available sources for online spaces and games for GIaNT children and youth.

## Introduction

Puberty is the process of physical changes initiated by hormonal signals from the brain to the gonads between the ages of 8 to 14 years old. Sexual health and puberty education is mandated across all provincial and territorial curricula in Canada. However, this information tends to be inadequate or outdated, failing to meet either international standards or the 2019 Canadian Guidelines for Sexuality Education [1]. Few resources provide adequate support for gender-independent, intersex, non-binary, or transgender (GIaNT) children and youth whose gender identity does not directly align with conventional visible markers of physiological sex and its medicalization. Gender identity and gender-affirmation are integral to one’s sense of well-being at all stages of development [2].

When considering puberty health education for GIaNT youth, it is crucial to understand that such youth’s puberty health needs are not the same as their cisgender counterparts [3–5]. Overwhelmingly, puberty and sexual education programs are not inclusive to gender-diverse youth [4, 5]. GIaNT youth have expressed not only a need for comprehensive and inclusive puberty health education programs but also the desire to explore such pathways in an online setting [4, 5]. Online spaces are generally preferred amongst GIaNT and other 2SLGBTQIA+ (Two Spirit, lesbian, gay, bisexual, transgender, queer, intersex, asexual, and “+” stands for orientations and identities not named in the initialism) youth because these are regarded as “safe,” meaning, they can undergo transformative, affirming, and individualized education as they see fit [5–7] without external limitations like unsupportive caretakers/parents, or homophobic and/or transphobic judgement or bias [5, 7]. Since most youth gain access to puberty health information online [5, 7], digital platforms appear to be a promising method for disseminating such educational programs [7–9].

One popular form of digital platforms is called role-play games. Role-play games (RPG’s) are a popular genre of video game that allow the player to control a character’s actions within the game. This usually means the player makes autonomous, personalized choices on where to go in the world, decide how to navigate through story options, as well as interact with the game world in other ways, such as with items, places, and other characters. RPG’s are typically user-friendly, do not require intricate hand-eye coordination, and are story/character-based games. RPG’s are widely sought-out by GIaNT youth because they permit exploration of one’s gender identity and expression through fictional characters, which is a crucial component for emotional intimacy, relatability, safeness, and relevance among them [7, 10].

An initial search of the literature shows that the majority of games and online resources for GIaNT youth and children focus on mental health [11, 12], which includes accompanying concerns of smoking cessation and/or addiction [8, 13, 14]. The possibility of using online gaming as a mental health intervention is a common concern we have found in our preliminary search on the topic [8, 12]. While mental health is, of course, encompassed in a holistic view of health (especially in the context of gender-diverse youth), it makes up only one form of health, and does not include a broader scope of puberty health needs for GIaNT youth. For example, some of the GIaNT youth participants in research studies have expressed that their only exposure to sexual education curriculums center issues like pregnancy and sexually transmitted infections, and only in the context of cisgender youth [13, 15]. Such curriculums are not applicable to the needs and desires that GIaNT youth express towards information on puberty health.

A preliminary search of MEDLINE, the Cochrane Database of Systematic Reviews and JBI Evidence Synthesis was conducted, and we found two current systematic reviews that are similar to our review topic but not focused on identified objective. These systematic reviews are: 1.) Effectiveness, acceptability, and feasibility of digital health interventions for LGBTIQ+ Young People: Systematic Review by Gilbey et al. [9], and 2.) Sex education in the spotlight: What is working? systematic review by Lameiras-Fernández et al. [16] While similar in subject matter, neither of these systematic reviews specifically focused on GIaNT youth. Particularly the former article focused on mental health interventions, and not necessarily a holistic view of puberty health needs. Lameiras-Fernández et al. explored the limitations and possibilities of casting out sexual education curricula in digital settings to youth but did not focus on GIaNT youth. Therefore, the aim of this scoping review is to address this gap in literature.

For these reasons, the purpose of this scoping review protocol will be to search for what has been reported on the development and the use of gender-affirming online resources and games for GIaNT children and youth. JBI scoping review methodology will be utilized to identify the existing literature for online resources and games for GIaNT youth, as well as investigate existing literature on specialized needs for GIaNT youth puberty health education. This method of inquiry is ideal for mapping and exploring the literature on a topic, as it draws from a broad range of study designs and evidence types to inform the findings.

### Review questions

What has been reported on the development and the use of gender-affirming online spaces and games for GIaNT children and youth? What has been reported on online spaces or games for GIaNT youth to address their puberty health needs?

### Inclusion criteria

The inclusion criteria for this scoping review are as follows: 1.) Participants/study must include GIaNT people. 2.) Participants/study must target children, youth, or young people within the age group of 9–26. 3.) Studies may include the use, implication, and/or discussion of gaming, online resources, or other digital platforms in relation to the population. Digital platforms may include online websites, apps, and social media. The exclusion criteria include individuals who are under 9 years of age and over 26 years of age, do not identify as GIaNT people, and sources that does not include games, online sources, or other digital platforms.

#### Population

Transgender refers to any individual whose gender identity does not align with their assigned-sex-at-birth [17]. It is often referred to as an “umbrella” term because it includes but is not limited to individuals who identify as: transgender women, transgender men, non-binary, gender-fluid, gender queer, and more [18, 19]. Intersex people are sometimes included in this category, but it is commonly anticipated amongst intersex and queer communities that such a label is determined on an individualized basis [20, 21]. In other words, not all intersex people align themselves with the transgender identity [17]. Non-binary refers to individuals who do not align their gender identities within the man/woman gender binary [20, 21]. This can include people who are agender, gender-fluid, or genderqueer. Some, but not all, individuals who are non-binary identify as transgender. Our team uses gender-independent and gender-diverse to refer to anyone who may be questioning their gender, or otherwise view themselves as not cisgender, but have not identified themselves within a specific gender identity.

We define youth as individuals between ages 9–26. This age range was based on an initial review of the literature. Although not explicitly explained, this seemingly older age range may be for three reasons: 1.) The potential use of (or desire to use) puberty blockers within the population; [10, 18] 2.) The possibility of a “second puberty,” which refers to GIaNT individuals who choose to use hormonal therapy, which initiates a “second puberty” process within the body for those who have already undergone puberty; [10, 18] and 3.) Considering the apparent pervasive lack of access to comprehensive puberty health needs, coupled with transgender information and options are not widely made available to youth. Moreover, many of the GIaNT individuals come to an understanding of their gender identities in a variety of timelines [10, 18, 22]. To this end, it is necessary to broaden our age range to include seemingly older individuals as youth.

#### Concept

This review will consider studies that examine online spaces or game development and its use for GIaNT youth.

#### Context

Sources will have a global context and will not be limited to geographical location or clinical care setting.

#### Types of studies

This scoping review will consider quantitative, qualitative, and mixed methods study designs for inclusion. In addition, systematic reviews, text, and opinion papers will be considered for inclusion in the proposed scoping review. The review of grey literature will not be limited to Canada’s professional sites, and it will consider key international websites and resources, such as educational and training resources for healthcare professionals for the purpose of puberty healthcare for GIaNT youth.

## Methods

The proposed scoping review will be conducted in accordance with JBI methodology for scoping review as evident in the JBI Manual for Evidence Synthesis [23].

### Search strategy

The search strategy will aim to locate both published and unpublished studies. An initial limited search of Web of Science and PubMed (MedLine) was undertaken to identify seed articles on the topic (Table 1).

**Table 1:** Search strategy.

Search	Query	Records retrieved
#1	Adolescen* or Child* or teen* or youth or "young adult" OR "young people" OR kids	3,574,280
#2	"sexual minorit*" OR "Sexual orientation" OR "gender minorit*" OR "gender identit*" OR "gender expression*" or "gender divers*" or "Questioning Persons" or "Gender Transition" or Transgender or non-binary or nonbinary or "gender diverse" or "gender minorit*" or agender or "gender queer" or genderqueer or bigender or LGBT* OR intersex OR 2SLGBTQIA+ OR "gender independent" OR "trans and nonbinary" OR "trans and non-binary" OR transsexual* OR "gender nonconforming" OR "gender non-conforming" OR "gender-nonconforming" OR GNC OR "trans men" OR "trans women" OR "trans people" OR “FTM” OR “female to male” OR “MTF” OR “male to female” OR SGMY OR "sexual and gender minorit*"	90,782
#3	OER OR "open educational resource*" OR "interact* media" OR "digital interven*" OR "serious gam*" OR MMO OR "massive multiplayer online" OR "educational gam*" OR "digital gam*" OR "Video Gam*" or gam* OR "internet resource*" OR "online resource*" OR "digital resource*" OR "role play gam*" OR RPG OR avatar* OR "online gam*" OR "game-based learning" OR "game element" OR "game utilization" OR "game based learning" OR "computer gam*" OR "internet-based intervent*"	1,657,631
#4	#1 AND #2 AND #3	383
No limitations.		

Web of Science, search conducted on June 20, 2023 at Toronto Metropolitan University Library

The text words contained in the titles and abstracts of relevant articles, and the index terms used to describe the articles will be used to develop a full search strategy for CINAHL, Cochrane, Epistemonikos, ERIC, Gender Studies Database, GenderWatch, LGBTQ+ Source, ProQuest, ProQuest Dissertation and Theses Global, PyscInfo, and Scopus. The search strategy, including all identified keywords and index terms, will be adapted for each included information source. To include relevant grey literature, no limits on the type of the sources is applied. The reference lists of all studies selected for critical appraisal will be screened for additional studies. There will be no language restriction since preliminary search results were mostly in English. Study type or publication date will not be restricted. Due to the limited number of sources in the literature, sources published on any date will be included.

### Study selection

Following the search, all identified citations will be collated and uploaded into Zotero and Covidence™, an online systematic review organization tool, and duplicates removed. Following a pilot test, titles and abstracts will then be screened by two independent reviewers for assessment against the inclusion criteria for the review. Two independent reviewers will screen, select, and extract eligible studies. In the title and abstract screening phase, Google Translate will be utilized for sources not in English. If a non-English source is moved forward to the full-text review, Google Translate will be utilized to extra data. If unable to extract data from the non-English sources or unable to check the accuracy of translation, these sources will be excluded, and this exclusion will be identified in the Prisma diagram.

Potentially relevant studies will be retrieved in full and Covidence will be utilized for management, assessment, and extraction of data. The full text of selected citations will be assessed in detail against the inclusion criteria by two independent reviewers. Reasons for the exclusion of sources of evidence in full text that do not meet the inclusion criteria will be recorded and reported in the scoping review. Any disagreements that arise between the reviewers at each stage of the selection process will be resolved through discussion, or with an additional reviewer/s. The results of the search and study selection and inclusion process will be reported in full in the final systematic review and presented in a Preferred Reporting Items for Systematic Reviews and Meta-analyses (PRISMA) flow diagram [24].

### Data extraction

A draft extraction tool is provided (Table 2). The draft data extraction tool will be modified and revised as necessary during the process of extracting data from each included paper. Modifications will be detailed in the full scoping review. Data will be extracted from studies included in the review by two independent reviewers using the standardized JBI data extraction tool. The data extracted will include specific details about the populations, study methods, interventions, and outcomes of significance to the review questions. Any disagreements that arise between the reviewers will be resolved through discussion or with a third reviewer. Authors of papers will be contacted to request missing or additional data, where required.

**Table 2:** Data extraction instrument.

Author(s) name(s)
Covidence will be used to reference the source (including DOI as relevant). Type of source: • Peer reviewed journal article • Dissertations and thesis • Educational materials for healthcare professionals • Discussion paper • Opinion piece • Commentary/editorial • Book chapter • Policy • Website (organization type)
Q1. Where did the study take place? (Alternatively, where do the majority of the authors reside?)
Q2. What were the participants’ age or the age focus of the source? • 9–26 years old
Q3. What was the focused population of the source? a) Gender independent/gender diverse b) Non-binary c) Transgender d) Intersex e) Other (Fill in the blank)
Q4. What were the online sources discussed in the source? • Open access resource • Social media • Apps • Gaming • Website • Other
Q5. What was the primary focus of the content on the online sources discussed in the source? • Puberty • Sex education • Gender-affirming • Gender exploration • Peer mentor • Peer support • Mental health • Smoking • Gender affirmation • Bullying • Suicide prevention
Q 6. What were the relevant findings to the review question: • Puberty health • Game development • Game usability and uptake • Online development sources • Online development source use • Gender-affirming • Age appropriate (9–26)
Q7. Was this a mixed method (included both quantitative and qualitative) study?
Q8. Did the authors use a validated instrument to generate quantitative outcomes data for the online source? If yes, proceed to Q. 9.
Q9. What validated instrument tool was used to collect quantitative data?
Q10. Did the authors report qualitative data in their research? If yes, please answer Q11 and Q12.
Q11. What methodology did the authors use to capture or collect the qualitative data they reported? Check all that apply or add another method if not listed. • Focus groups • Key informant interviews • Written reflection/reflective writing • Written responses to open-ended questions • Direct observation of learners or teams • Not adequately described. • Other
Q12. What methodological approach did the authors use to analyze qualitative data? Check all that apply, or specify the methodology used if not listed. • Content analysis • Narrative analysis • Discourse analysis • Framework analysis • Grounded theory • Thematic analysis • Not adequately described. • Other
Q13. What did the authors identify as valuable for GIaNT Youth and children? (If cut and paste, use quotes).
Q14. What challenges or limitations did the authors find? (If cut and paste, use quotes).
Q15. What take-home points, if any, did you take from this article?
Q16. What challenges or limitations did you (as the reviewer) identify in this source?

### Data analysis and presentation

The extracted data will be presented in tubular format (Table 2). In addition to charted results, a narrative summary will describe how the results respond to the review questions.

## Supporting information

S1 Checklist PRISMA-P (Preferred Reporting Items for Systematic review and Meta-Analysis Protocols) 2015 checklist: Recommended items to address in a systematic review protocol*. (DOC)
